# Analysis of tag-position bias in MPSS technology

**DOI:** 10.1186/1471-2164-7-77

**Published:** 2006-04-07

**Authors:** Junfeng Chen, Magnus Rattray

**Affiliations:** 1 School of Computer Science, University of Manchester, Manchester, UK

## Abstract

**Background:**

Massively Parallel Signature Sequencing (MPSS) technology was recently developed as a high-throughput technology for measuring the concentration of mRNA transcripts in a sample. It has previously been observed that the position of the signature tag in a transcript (distance from 3' end) can affect the measurement, but this effect has not been studied in detail.

**Results:**

We quantify the effect of tag-position bias in Classic and Signature MPSS technology using published data from Arabidopsis, rice and human. We investigate the relationship between measured concentration and tag-position using nonlinear regression methods. The observed relationship is shown to be broadly consistent across different data sets. We find that there exist different and significant biases in both Classic and Signature MPSS data. For Classic MPSS data, genes with tag-position in the middle-range have highest measured abundance on average while genes with tag-position in the high-range, far from the 3' end, show a significant decrease. For Signature MPSS data, high-range tag-position genes tend to have a flatter relationship between tag-position and measured abundance. Thus, our results confirm that the Signature MPSS method fixes a substantial problem with the Classic MPSS method. For both Classic and Signature MPSS data there is a positive correlation between measured abundance and tag-position for low-range tag-position genes. Compared with the effects of mRNA length and number of exons, tag-position bias seems to be more significant in Arabadopsis. The tag-position bias is reflected both in the measured abundance of genes with a significant tag count and in the proportion of unexpressed genes identified.

**Conclusion:**

Tag-position bias should be taken into consideration when measuring mRNA transcript abundance using MPSS technology, both in Classic and Signature MPSS methods.

## Background

A number of high-throughput technologies have been developed that are able to measure the abundance of many mRNA transcripts within a sample. These include microarray technology[1,2], SAGE (Serial Analysis of Gene Expression) technology[3,4] and most recently MPSS (Massively Parallel Signature Sequencing) technology[5,6]. Compared with microarray technology, SAGE and MPSS technologies have some clear advantages. In these tag-based technologies, transcript abundance is measured by counting signature sequences and there is no need to identify in advance the set of target transcripts. For most microarray technologies, the set of potential targets must be available in advance so that appropriate probe sequences can be used. Moreover, microarrays are sensitive to cross-hybridization noise, which limits their ability to detect transcripts with low abundance. Compared with both microarray and SAGE technologies, MPSS is more sensitive and can be used to reliably measure weakly expressed transcripts at concentrations as low as 5 tpm (transcript per million), while SAGE is restricted to measure concentrations of 100 tpm or more[7]. MPSS is therefore one of the most powerful and promising new technologies for the quantitative analysis of gene expression. It has the potential to determine the relative concentration of almost all mRNA molecules within a cell population and has already been used for expression analysis in several organisms[8,9]. An interesting feature of this technology, which differentiates it from microarrays, is that it can be more credibly used to study the relative expression level of different genes within a sample. This is difficult to do with microarrays, because sequence-specific effects result in huge variations in binding affinity for different microarray probes and the signal associated with each probe cannot be considered a measure of relative abundance of different genes within a sample. Using microarrays, one can only accurately measure the relative abundance of the same gene between samples while tag-based technologies are thought to provide a better measurement of absolute abundance. MPSS and SAGE data have therefore been useful for studying general features of expression level that are more difficult to assess using microarray data[10,11]. There exists two basic MPSS methods: one is the original Classic MPSS method and the other is the more recently developed Signature MPSS method. The difference between these methods is that for the Classic method, the entire fragment from the Sau3A (GATC, or DpnII) site to the poly(A) is cloned and loaded onto the beads for sequencing. In the Signature method, during cloning, a MmeI enzyme recognition site is added to cut 21 or 22 bp from the recognition site for sequencing. The Signature method is intended to remove any bias during the bead loading or sequencing reactions that may result from different DpnII-to-poly(A) fragment sizes[12]. Although MPSS technology has enormous advantages, as described above, it also has its own associated deficiencies. Firstly, not all genes can be identified using MPSS technology. Genes without Sau3A (DpnII) sites cannot be detected. Secondly, Meyers MPSS lab  has pointed out that, for Classic MPSS data, if there is an unusually long distance (e.g. > 800 bp) between the 3' end of the transcript (poly(A) site) and the first Sau3A (DpnII) site, then these genes may not appear in the library. A recent study of human gene expression using Classic MPSS data found that tags with this distance greater than 300 bp are more than threefold less abundant on average than those with lower distances[8]. That means there is a tag-position bias in MPSS technology. However, until now no detailed analysis of this tag-position bias has been reported. In this paper, our purpose is to investigate the tag-position bias in both Classic and Signature MPSS data and to quantify its impact. We define tag-position to be the distance between the 3' end of a transcript and the 3' most Sau3A (DpnII) site. Our approach is to investigate the dependence between the measured gene expression level and tag-position on a genome-wide scale. To do that, we use nonlinear regression techniques. There are many gene structure features that are known to be correlated with gene expression levels, such as mRNA length and number of exons[10]. We therefore also compare these influences with tag-position bias to evaluate the relative size of the effect of tag-position bias in MPSS data analysis.

## Results

We obtain MPSS data sets from the Meyers lab[13,14] and the Ludwing Institute for Cancer Research (LICR)[15], including Arabidopsis Classic and Signature data, Rice Signature data and Human Classic data. Through data preparation and preprocessing steps (see Methods), we mapped tags to transcripts and selected genes for analysis. The complete list of these genes and related parameters are in the Supplementary File. [see Additional file 1]

### Relationship between tag-position and measured expression level

**Figure 1:**
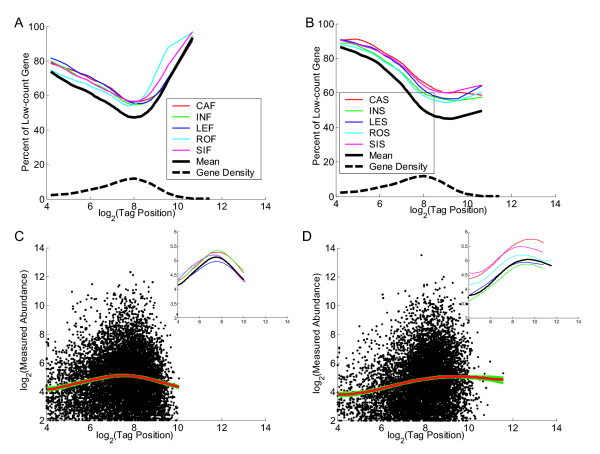
**Tag-position bias analysis using Classic and Signature data from Arabidopsis**. Plots A and B show the relationship between tag-position and the percentage of low-count genes. The gene density shows the distribution of genes with a given tag-position. The curves on the top of each plot show the relationship between tag-position and the percentage of low-count genes. Plots C and D show the dependence of measured abundance on tag-position for genes with significant associated tag-count. The scatter-plot shows all data points from five different libraries. The red curve represents a LOWESS smooth of all these data and the green curve represents the bootstrap credibility intervals (5–95%). (Inset) Each line except the bold black one represents a LOWESS smooth of an individual library, while the bold black one is the same as the red one in the main plot. Plots A and C are from Classic MPSS data while Plots B and D are from Signature MPSS data.

**Figure 2:**
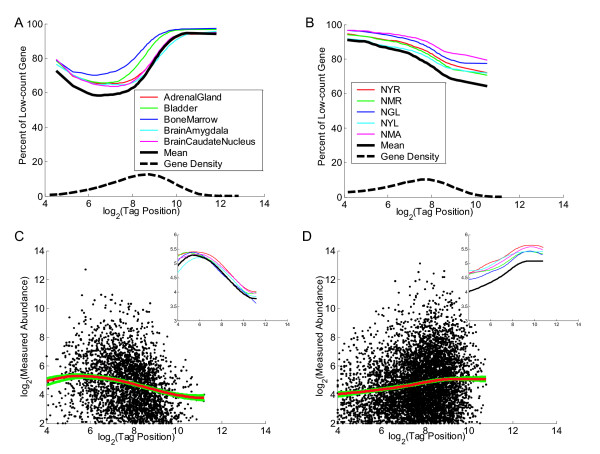
**Tag-position bias analysis using Human Classic and Rice Signature data**. The same as described in Figure 1 except using Human Classic Data (Plots A and C) and Rice Signature Data (Plots B and D).

Firstly, we investigate the relationship between tag-position and measured RNA abundance using Classic and Signature MPSS data from Arabidopsis. We adopted two different methods to analyse and visualise the relationship (Fig 1). One method is to calculate the percentage of low-count genes (genes with count < 4 tpm) for different tag-position ranges. From Fig 1A and Fig 1B, we see that Classic and Signature MPSS data show different patterns. Classic MPSS data shows a greater percentage of low-count genes in the high tag-position range while Signature MPSS data shows a flatter relationship. Both Classic and Signature MPSS data show a constant decrease in the percentage of low-count genes in the low tag-position range. The other method is to only consider genes with significantly measured abundance (genes with count ≥ 4 tpm) and apply locally weighted scatter-plot smoothing (LOWESS, a nonlinear regression method) to investigate the relationship between tag-position and measured RNA abundance. From Fig 1C and Fig 1D, we see that Classic and Signature MPSS data again show different patterns. Classic MPSS data tends to decrease in the high tag-position range while Signature MPSS data shows little change. Both Classic and Signature MPSS data show an increase in the low tag-position range. The dynamic range of the mean measured abundance is more than twofold, although this is really an underestimate of the effect since this excludes those genes with zero tag-count identified in Fig 1A and Fig 1B. We used Human Classic and Rice Signature data to confirm the result from Arabidopsis Classic and Signature data. In Fig 2 the left column shows the Human data and the right column shows the Rice data with the plots otherwise corresponding to those in Fig 1. We see that the results are broadly similar. One difference is that Human Classic MPSS data seems to behave differently in the low tag-position range. This is probably mainly because Human has more genes with high tag-position and less with low tag-position, as shown in the gene density curve of Fig 2A which is shifted to the right.

### Comparison of Classic and Signature MPSS data from the same sample

**Figure 3:**
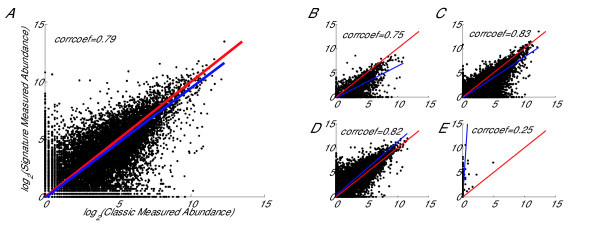
**Comparison with measured abundance using Classic and Signature data from Arabidopsis**. Plots compare the measured abundance using Classic and Signature data from Arabisopsis. In Plot A, all data points in the scatter-plot represent mean values of 21608 genes from Classic Data and Signature Data of five experiments measuring the same sample. The red line is the middle line (x = y) and the blue line is calculated by total least squares. In Plots B-E, genes are selected with *log*_2 _(tag-position) in the ranges [4 6], [6 8], [8 10] and [10 12], with 3417, 10005, 8037 and 149 genes in each range respectively.

We have confirmed that there is a significant tag-position bias which affects both Classic and Signature MPSS data. Here, using Classic and Signature MPSS data from same samples of Arabidopsis, we compare and visualise the difference in their measured abundance (Fig 3). From Fig 3A, we observe that many data points are far from the middle line, which indicates that the differences in measured abundance between Classic and Signature MPSS are significant. By selecting different tag-position ranges, as shown in Fig 3B–E, we can clearly see some difference between the two MPSS methods. As a whole, Classic MPSS data tends to have higher measured abundance in the low tag-position range; but, with the increase of tag-position, measured abundance of Signature MPSS data tends to increase quickly and is much higher in the high tag-position range. This result supports our previous observations about the relationship between measured expression level and tag-position.

### Comparison with effects from mRNA length and exon number

**Figure 4:**
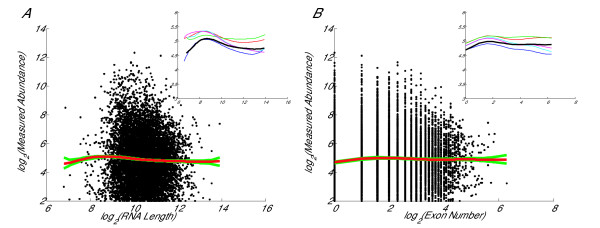
**Dependence of expression on mRNA length and number of exons**. Plot A shows the dependence of measured abundance on mRNA length. Plot B shows the dependence of measured abundance on number of exons.

Although we observe the effect due to tag-position, there exist many other factors which could affect gene expression level. Here we compare with the effects from mRNA length and exon number, which were found to be significant factors that affect gene expression by Chiaromonte et al[10]. From Fig 4A, we see that there exists a slight negative linear relationship between expression level and mRNA length. From Fig 4B, we see that there also exists a mostly negative correlation between expression level and number of exons with a positive correlation at low exon numbers. However, comparison with the tag-position effect illustrated in Fig 1 shows that tag-position bias is a much more significant effect than these two features. We initially suspected that some of the trend observed between tag-position and measured abundance might relate to a correlation between tag-position and these features, eg. high tag-position is more likely for genes with long transcripts. These weak trends suggest this is unlikely to be the case.

## Discussion

With the development of gene expression analysis technologies, many studies have been focused on discovering factors that affect gene expression levels. Some studies have looked at gene structure factors such as length of gene, length of mRNA, number of exons and distance between genes on the genome, which may genuinely affect expression level[10]. Other studies, like this one, focus on those biases existing in the experimental technologies themselves[16]. MPSS technology has some great advantages as a method for measuring mRNA transcript abundance. It is readily allows identification of most of a cell's rarely expressed mRNA and it has been successfully used to analyze gene expression for many different organisms. Therefore it is important to consider technical biases and deficiencies that should be taken into consideration. One problem is that a small percentage of of MPSS tags could be mapped to more than one part of the genome. Another problem is that some mRNA transcripts without Sau3A (DpnII) sites cannot be analyzed. Also, as confirmed in this paper, mRNA transcripts having long distances between the 3' end and the first Sau3A (DpnII) sites may not be detected in the Classic method. We have analysed the association between measured RNA abundance and tag-position. Using data sets from both Classic and Signature methods, from Arabidopsis, Rice and Human, our analysis indicates that there exist consistent tag-position biases in both methods. This bias is greater than the effect of morphological parameters described by Chiaromonte et al[10]. In fact, the only factor considered from that study with a comparable effect was the length of mRNA transcript, while exon number has a much weaker influence than tag-position. Tag-position bias therefore plays an important role in determining the tag count in MPSS technology and should not be disregarded when analysing mRNA abundance. We think that the main reason for the tendency of decrease of measured abundance of Classic data and flatter measured abundance of Signature data in the high tag-position range is that PCR amplification is inefficient for longer sequences. Indeed, the Signature method was originally designed to solve this problem and our results confirm that the Signature MPSS method fixes the main problem with the Classic MPSS method. However, the reason that both Classic and Signature data have a tendency towards increasing measured abundance with tag-position in the low tag-position range is unclear. The nonlinear regression curves, obtained by LOWESS, could be used to normalise tag-count in order to correct for this bias. However, little can be done to correct for missing or very low tag counts and this is an especially big problem for genes with high tag-positions in the Classic method or low tag-positions under both methods.

## Conclusion

Our analysis reveals that there exists significant tag-position bias in both Classic and Signature MPSS data. We confirm that, in the Classic MPSS method, the tags which are far from the 3' end are associated with relatively low tag-counts on average and as the distance increases they are increasingly likely to have very low or zero tag-count. We also found that, in both Classic and Signature MPSS methods, the tags which are closer to the 3' end are associated with relatively low tag-count and an increased likelihood of zero tag-count. Our findings confirm that tag-position has an important influence in MPSS technology and we argue that this effect should be taken into consideration when measuring mRNA transcript abundance using MPSS technology, both in the Classic and the Signature MPSS methods. For example, statistical regression methods applied to MPSS data could include tag-position as an additional regression variable in order to reduce bias.

## Methods

### Data preparation and preprocessing

We downloaded Arabidopsis and Rice MPSS data from the Meyers lab[13,14], which include 10 libraries for Arabidopsis (5 Classic and 5 Signature) and 5 libraries for Rice (all Signature). We obtained Human MPSS data from LICR[15] and selected 5 libraries from different tissues (all Classic). We downloaded Arabidopsis genome release data (November 2005) from TAIR[17], Rice genome release data (December 2004) from TIGR[18] and Human genome release data (October 2004) from NCBI[19]. From the genome data files, we extracted annotated genes, transcripts and genome sequence. Genome and transcriptome data were constructed using genome sequence and annotated transcripts. We extracted all possible MPSS tags from the genome and transcriptome, and combine them together to get the tag's mapping relationship with genes. We calculated morphological parameters of genes and transcripts, including mRNA length and number of exons. The next step was to select genes for our analysis. Firstly, we selected protein-coding genes that were associated with only one type of annotated transcript, to avoid ambiguity due to splice variants. Then we obtained all the 3' most tags in the transcriptome, checked if they were unique in the transcriptome mapping, and discarded those genes with non-unique 3' most tags. Repeating this in different genomes, we obtained 21608 Arabidopsis genes, 30214 Rice genes and 10680 Human genes for investigation. We then calculated all tag-positions as the distance from the poly(A) to the 3' most tag in the transcript.

### Data analysis

We used scatter-plots to represent the data and data were log_2_-transformed to aid visualisation. In the analysis, 4 tpm is an important indicator for significant measured RNA abundance, since this is believed to be the lower limit of current MPSS technology[12]. In this paper, we define low-count genes as genes with measured abundance smaller than 4 tpm and significantly measured genes as genes with measured abundance greater than and equal to 4 tpm. We used regression methods to analyse the data. Because the relationship between two variables was often non-linear, we used local weighted scatter-plot smoothers (LOWESS)[20] to get smooth curves. This curve showed the dependence pattern between two variables on the scatter-plot. When using LOWESS, we used a Gaussian kernel to calculate the weight contributing to each point from its neighbor points. We also used the bootstrap method[21] to evaluate the credibility intervals (5–95%) of the LOWESS method. When comparing the Classic and Signature MPSS using same samples, we selected Arabidopsis data from the Meyers lab[13], include 5 different tissues with their individual Classic and Signature libraries names as CAF/CAS (Callus), INF/INS (Infloresence), LEF/LES (Leaves), ROF/ROS (Root) and SIF/SIS (Silique). A Total Least Square method[22] was used to show the linear regression relationship between Classic and Signature MPSS expression data in various tag-position ranges.

## Authors' contributions

JC performed the analysis, evaluated the results and drafted the manuscript. MR initiated the project, helped with evaluation of the results and manuscript preparation, and provided mentoring. Both authors read and approved the final manuscript.

## Supplementary Material

Additional File 1 Excel file includes lists of selected 21608 genes for Aradisopsis, 30214 genes for Rice and 10680 genes for Human used for investigation, including the features used in this paper. Click here for file
